# Uptake of ^68^gallium in atherosclerotic plaques in LDLR^-/-^ApoB^100/100 ^mice

**DOI:** 10.1186/2191-219X-1-14

**Published:** 2011-08-17

**Authors:** Johanna MU Silvola, Iina Laitinen, Henri J Sipilä, V Jukka O Laine, Pia Leppänen, Seppo Ylä-Herttuala, Juhani Knuuti, Anne Roivainen

**Affiliations:** 1 Turku PET Centre, University of Turku and Turku University Hospital, Turku, Finland; 2 Nuklearmedizinische Klinik der Technischen Universität München, Munich, Germany; 3 Department of Pathology, Turku University Hospital, Turku, Finland; 4 A. I. Virtanen Institute for Molecular Sciences, University of Eastern Finland, Kuopio, Finland; 5 Turku Center for Disease Modeling, University of Turku, Turku, Finland

**Keywords:** atherosclerosis, plaque, autoradiography, gallium-68, PET

## Abstract

**Background:**

Atherosclerosis is a chronic inflammatory disease of artery wall characterized by infiltration of monocytes into subendothelial space and their differentiation into macrophages. Since rupture-prone plaques commonly contain high amounts of activated macrophages, imaging of the macrophage content may provide a useful tool for the evaluation of plaque vulnerability. The purpose of this study was to explore the uptake of ^68^gallium (^68^Ga) in atherosclerotic plaques in mice.

**Methods:**

Uptake of ionic ^68^Ga was investigated in atherosclerotic LDLR^-/-^ApoB^100/100 ^and C57BL/6N control mice at 3 h after injection. The *ex vivo *biodistribution of the ^68^Ga was assessed and autoradiography of aortic cryosections was defined. *In vivo *imaging of ^68^Ga was performed using a small animal positron emission tomography PET/CT scanner.

**Results:**

Our results revealed that the uptake of ^68^Ga-radioactivity was higher in atherosclerotic plaques than in healthy vessel wall (ratio 1.8 ± 0.2, *p *= 0.0002) and adventitia (ratio 1.3 ± 0.2, *p *= 0.0011). The autoradiography signal co-localized with macrophages prominently as demonstrated by Mac-3 staining. In both mice strains, the highest level of radioactivity was found in the blood.

**Conclusions:**

We observed a moderate but significantly elevated ^68^Ga-radioactivity uptake in the aortic plaques of atherosclerotic mice, especially at the sites rich in macrophages. While the uptake of ^68^Ga was promising in this animal model, the slow blood clearance may limit the usability of ^68^Ga as a PET tracer for clinical imaging of atherosclerotic plaques.

## Background

Atherosclerosis is a chronic inflammatory disease of artery wall with infiltration of monocytes into subendothelial space and their differentiation into macrophages. Macrophages express pro-inflammatory and chemotactic molecules thus playing an essential role in the formation, progression, and pathogenicity of atherosclerotic plaques. The population of macrophages in atherosclerotic lesions is heterogeneous. The majority of macrophages transform into foam cells by accumulating lipids in their cytoplasm, while other macrophages may have vascular protective and repair properties. Furthermore, some macrophages may secrete enzymes that degrade the extracellular matrix and fibrous cap, possibly leading to a rupture of the atherosclerotic plaque. Significantly higher quantities of macrophage-rich plaque areas have been reported in atherosclerotic patients with acute coronary syndromes than in patients with stable angina. Since the rupture-prone plaques commonly contain high amounts of activated macrophages, imaging of the macrophages may provide a useful tool for determining plaque vulnerability [1-5].

^68^Gallium (^68^Ga, *T*_1/2 _68 min) is a generator-produced, positron-emitting radionuclide with a high positron yield (89%) possessing suitable physical properties for *in vivo *PET imaging. ^67^Gallium (^67^Ga) has traditionally been used to image inflammation with gamma cameras. Since ^68^Ga owns the same chemical characteristics as ^67^Ga, it is assumed that ionic ^68^Ga could accumulate at the sites of macrophage-rich areas in inflammatory lesions. Furthermore, ^68^Ga-based PET may have some added advantages compared to ^67^Ga-based SPECT, such as a better resolution for the detection of inflammatory plaques [6]. The aim of this study was to determine the uptake of ionic ^68^Ga in the atherosclerotic plaques in mice.

## Methods

### Production of ionic ^68^Ga

^68^Ga radionuclide is readily available by elution from a ^68^Ge/^68^Ga generator (Cyclotron Co., Obninsk, Russia) with 0.1 M HCl. The radioactive elution peak, monitored online with a positron-sensitive photodiode detector (Hamamatsu S5591, Hamamatsu Photonics K.K. Solid State Division, Hamatsu City, Japan), was collected and the ^68^Ga-chloride was neutralized with 1 M NaOH to give pH value of 7, as described earlier [7]. The quantity of colloidal ^68^Ga in the final product was measured by ultrafiltration, as indicated by the activity retained on the 0.2- μm ultrafilter. The final ^68^Ga product contained 13% of colloidal forms of ^68^Ga.

### *Ex vivo *biodistribution of ^68^Ga

Nine 10-11-months-old male atherosclerotic LDLR*^-/-^*ApoB^100/100 ^mice (weight 35 ± 5 g, mean ± SD) [8,9] and six 2.5-month-old male non-atherosclerotic C57BL/6N control mice (weight 28 ± 1 g) were *ex vivo *investigated. LDLR*^-/-^*ApoB^100/100 ^mice were kept on a high-fat, Western-type diet (TD 88173, Harlan Teklad) for 3-4 months, starting at 7 months of age. C57BL/6N mice were fed with regular diet. The experiments were approved by the Laboratory Animal Care and Use Committee of the University of Turku, Finland.

^68^Ga (17 ± 2 MBq) was intravenously administered via a tail vein in non-anesthetized mice. Three hours after injection the mice were sacrificed under deep isoflurane anesthesia by cervical dislocation. Samples of blood and various tissues were excised, patted dry, weighed, and measured for radioactivity using an automatic gamma counter (1480 Wizard 3" Gamma Counter; EG & G Wallac, Turku, Finland) cross-calibrated with a dose calibrator (VDC-202, Veenstra Instruments, Joure, The Netherlands). The radioactivity remaining in the tail was compensated, background counts were subtracted, and the radioactivity decay was corrected to the time of injection. The concentration of the radioactivity accumulated in the various tissues over the 3-h period following ^68^Ga injection was expressed as a percentage of the injected activity per gram of tissue (% IA/g).

### Autoradiography analysis of aortic cryosections

The distribution of ^68^Ga-radioactivity in the aortic tissue was studied by combining digital autoradiography and hematoxylin and eosin (HE) staining. The aorta and internal control muscle sample from the same animal were frozen and sequential longitudinal 8- and 20- μm sections were cut with a cryomicrotome at -15°C, thaw-mounted onto microscope slides. The sections were air dried for 5 min and apposed to an imaging plate (Fuji Imaging Plate BAS-TR2025, Fuji Photo Film Co., Ltd., Japan). After an exposure time of 2.5 h, the imaging plates were scanned with Fuji Analyser BAS-5000 (Fuji Tokyo, Japan; internal resolution 25 μm). The 20- μm cryosections were stained with HE and examined for morphology under a light microscope. After carefully co-registration of the autoradiographs and HE images, ^68^Ga-radioactivities were measured in the following regions of interest (ROIs): (1) plaque (excluding media), (2) healthy vessel wall (no lesion formation), (3) adventitia (including adjacent fat), and (4) muscle (internal control tissue). In control mice, no plaque areas were seen and ROIs were defined for healthy vessel wall, adventitia, and muscle. In each mouse, the uptakes of ^68^Ga-radioactivities in the regions of interest were calculated and normalized against internal control tissue (muscle). The results of digital autoradiography analysis were expressed as photostimulated luminescence per square millimeter (PSL/mm^2^).

### Assessment of degree of inflammation in atherosclerotic plaques

Immunohistochemical staining with the mouse macrophage specific antibody (Mac-3, clone m3/84, BD Pharmingen, BD550292, USA - BD Biosciences Pharmingen, Franklin Lakes, NJ, USA) was performed using aortic longitudinal 8- μm cryosections in order to detect activated macrophages, as described earlier [10]. Based on the Mac-3 staining, plaque areas were divided into four categories for the semiquantitative estimation of the degree of plaque inflammation; (1) no inflammation = no macrophages, (2) mild inflammation = occasional macrophages, (3) moderate inflammation = occasional and some groups of macrophages, (4) severe inflammation = abundant infiltration of macrophages. The uptake of ^68^Ga-radioactivity in the different categories was studied with digital autoradiography from 8- μm cryosections combined with images of Mac-3 immunostained sections. The results of digital autoradiography analysis were normalized against internal control tissue (muscle) and expressed as PSL/mm^2^.

### *In vivo *imaging

Three additional female LDLR*^-/-^*ApoB^100/100 ^mice, aged 14 to 15 months and kept on a high-fat, Western-type diet for 5 to 6 months (weight 36 ± 10 g, mean ± SD) were *in vivo *imaged with the Inveon small animal PET/CT scanner (Siemens Medical Solutions, Knoxville, TN, USA) at 3 h after injection of ^68^Ga (16 ± 3 MBq). PET images were acquired for 15 min, followed by CT angiography (10 min) without moving the animal. To obtain vascular contrast, 0.2 ml of iodinated intravascular contrast agent eXIATM160XL (Binitio Biomedical Inc, Ottawa, ON, Canada) was injected. CT acquisition consisted of 270 projections acquired with exposure time of 400 ms, x-ray voltage of 80 kVp, and anode current of 400 μA for a full 360° rotation. Mice were kept fully sedated with 1.5% isoflurane during imaging. PET image was reconstructed using OSEM3D algorithm. The resulting matrix was 128 × 128 pixels with 159 transverse slices in a transaxial field of view of 12.7 cm (pixel size 0.87 × 0.87 × 0.80 mm). CT image was reconstructed using a modified Feldkamp algorithm. The resulting matrix was 256 × 256 pixels with 384 transverse slices (pixel size 0.17 × 0.17 × 0.17 mm). Co-registration of PET and CT images was done using an automatic weighted mutual information algorithm and confirmed visually on the basis of anatomical landmarks. Quantitative PET analysis was performed by drawing ROIs of the same size in the left ventricle (blood pool), aortic arch, and brachiocephalic artery as identified on the basis of the CT angiography by using the Inveon Research Workplace software (Siemens Medical Solutions, Knoxville, TN, USA). Radioactivity concentrations were corrected for injected radioactivity, and the results were expressed as percentage of injected radioactivity per gram of tissue (% IA/g) assuming 1 mL equals 1 g.

### Statistical methods

All the results are expressed as mean ± SD. Student's *t-*test and one-way ANOVA were applied when comparing the biodistribution results of the atherosclerotic and control mice. *T *test with a mixed Dunnett's model was used to correct the *p *values of biodistribution of aortic uptake versus different tissue uptakes in the atherosclerotic and control mice groups. In the autoradiographic analysis, a mixed model with Tukey-Kramer corrected *p *values was applied to individual mean values of ROIs. A *p *value less than 0.05 was considered as statistically significant.

## Results

### Characterization of atherosclerotic plaques

After 3 to 4 months on a Western-type diet, the LDLR*^-/-^*ApoB^100/100 ^mice had developed areas covered with atherosclerotic plaques in aortic arch, aortic arteries, and also in the distal part of aorta. Histologically, the plaques were of fibroatheroma type and some of them contained calcifications. In the aortas of the control mice, no atherosclerotic plaques were detected.

### *Ex vivo *biodistribution of ^68^Ga-radioactivity

The biodistribution of ^68^Ga-radioactivity at 3 h after i.v. injection is shown in Table 1. The highest level of ^68^Ga-radioactivity was found in the blood in both mouse strains. The uptake in the whole aorta was higher in LDLR*^-/-^*ApoB^100/100 ^mice than in control mice, but the difference did not reach statistical significance. The aorta/heart and aorta/blood uptake ratios were 1.6 ± 0.7 and 0.2 ± 0.1 in atherosclerotic mice and 1.2 ± 0.2 and 0.2 ± 0.1 in control mice, respectively. The measured radioactivities in the bone and liver of atherosclerotic mice were significantly lower than in the control mice.

**Table 1:** *Ex vivo *biodistribution of radioactivity in atherosclerotic LDLR*^-/-^*ApoB^100/100 ^and C57BL/6N control mice

	**LDLR**^***-/-***^**/ApoB**^**100/100**^ (*n *= 9, urine *n *= 8)	C57BL/6N (*n *= 6, urine *n *= 5)	*p *value
Aorta	1.99 ± 0.73	1.52 ± 0.40	NS^a^
Bladder	2.50 ± 0.62	1.92 ± 0.29	NS
Blood	8.60 ± 1.29	8.07 ± 0.55	NS
Bone	2.25 ± 0.50	4.08 ± 0.49	< 0.05
Fat	0.46 ± 0.21	0.51 ± 0.07	NS
Heart	1.26 ± 0.26	1.29 ± 0.20	NS
Intestine	2.71 ± 0.70	3.16 ± 0.36	NS
Kidney	4.07 ± 0.78	4.25 ± 0.33	NS
Liver	2.69 ± 1.14	8.34 ± 4.33	< 0.05
Lungs	3.28 ± 1.10	3.58 ± 0.33	NS
Lymph nodes	2.03 ± 0.51	2.35 ± 0.34	NS
Muscle	0.71 ± 0.24	0.71 ± 0.08	NS
Pancreas	1.21 ± 0.25	1.48 ± 0.21	NS
Spleen	3.20 ± 1.28	4.51 ± 1.63	NS
Thymus	1.89 ± 0.96	1.18 ± 0.15	NS

At 3 h after intravenous injection of ionic ^68^Ga. Results are expressed as % IA/g (mean ± SD). NS, not significant. ^a^Difference between atherosclerotic and control mice.

### Digital autoradiography of aortic cryosections

**Figure 1:**
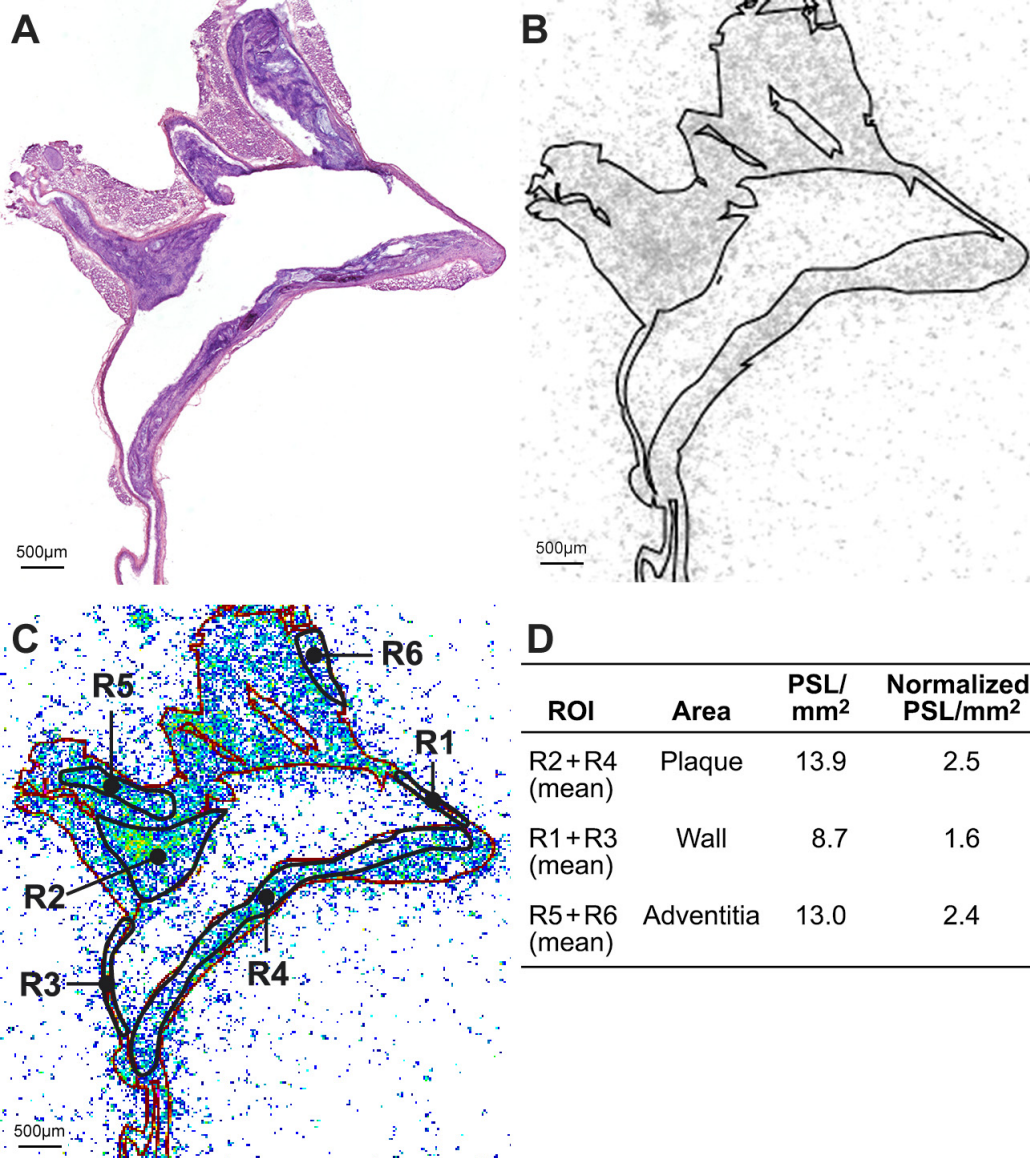
**An example of *ex vivo *aortic autoradiographic (ARG) analysis**. (**A**) HE staining of a 20-μm cryosection, showing the aortic arch and branches of arteries (**B**) autoradiograph (grayscale) from section (A) superimposed with the patch image made from the HE-stained section (A). (**C**) Autoradiograph superimposed with contour image and an example of ROI analysis: R2 and R4, plaque; R1 and R3, healthy vessel wall; R5 and R6, adventitia. (**D**) Panel shows the ARG results of section (C). Results are expressed as PSL/mm^2 ^(mean ± SD), normalized against the internal control tissue (muscle). In this representative section, the plaque/wall ratio is 1.6 and plaque/adventitia ratio 1.1, whereas in the whole study population, the corresponding ratios were 1.8 ± 0.2 and 1.3 ± 0.2.

A total of 8 to 12 autoradiographs of aortic cryosections from eight LDLR*^-/-^*ApoB^100/100 ^mice (one sample was discarded from the analysis because of remaining blood clot inside the aorta) and 8 autoradiographs of aortic sections from six control mice were generated and superimposed on corresponding digitalized HE staining images. In total, 1,208 ROIs (plaque *n *= 384, healthy vessel wall *n *= 396, adventitia *n *= 406, muscle *n *= 56) were analyzed from 14 mice. The autoradiography analysis revealed a significantly higher uptake of ^68^Ga-radioactivity in plaques compared with healthy vessel wall (3.4 ± 0.6 vs. 1.9 ± 0.3 PSL/mm^2^, *p *= 0.0002) and adventitia (3.4 ± 0.6 vs. 2.6 ± 0.3 PSL/mm^2^, *p *= 0.0011). The plaque/wall ratio was 1.8 ± 0.2 and plaque/adventitia ratio 1.3 ± 0.2 in LDLR*^-/-^*ApoB^100/100 ^mice (Figure 1). The difference between the adventitia and the healthy vessel wall was not statistically significant in either of the mouse strains. A relatively high uptake of ^68^Ga-radioactivity was detected in the calcified areas (*n *= 55 from the total 384 plaque ROIs) of atherosclerotic plaques vs. healthy vessel wall (ratio 2.1 ± 0.6, *p *= 0.0010). Calcified areas did not contain any macrophages. No statistically significant difference was detected in calcified areas of plaques vs. non-calcified plaques. The variability of the used digital autoradiography analysis has been tested and described earlier [10].

### Assessment of ^68^Ga-radioactivity uptake in macrophage-rich areas of atherosclerotic plaques

**Figure 2:**
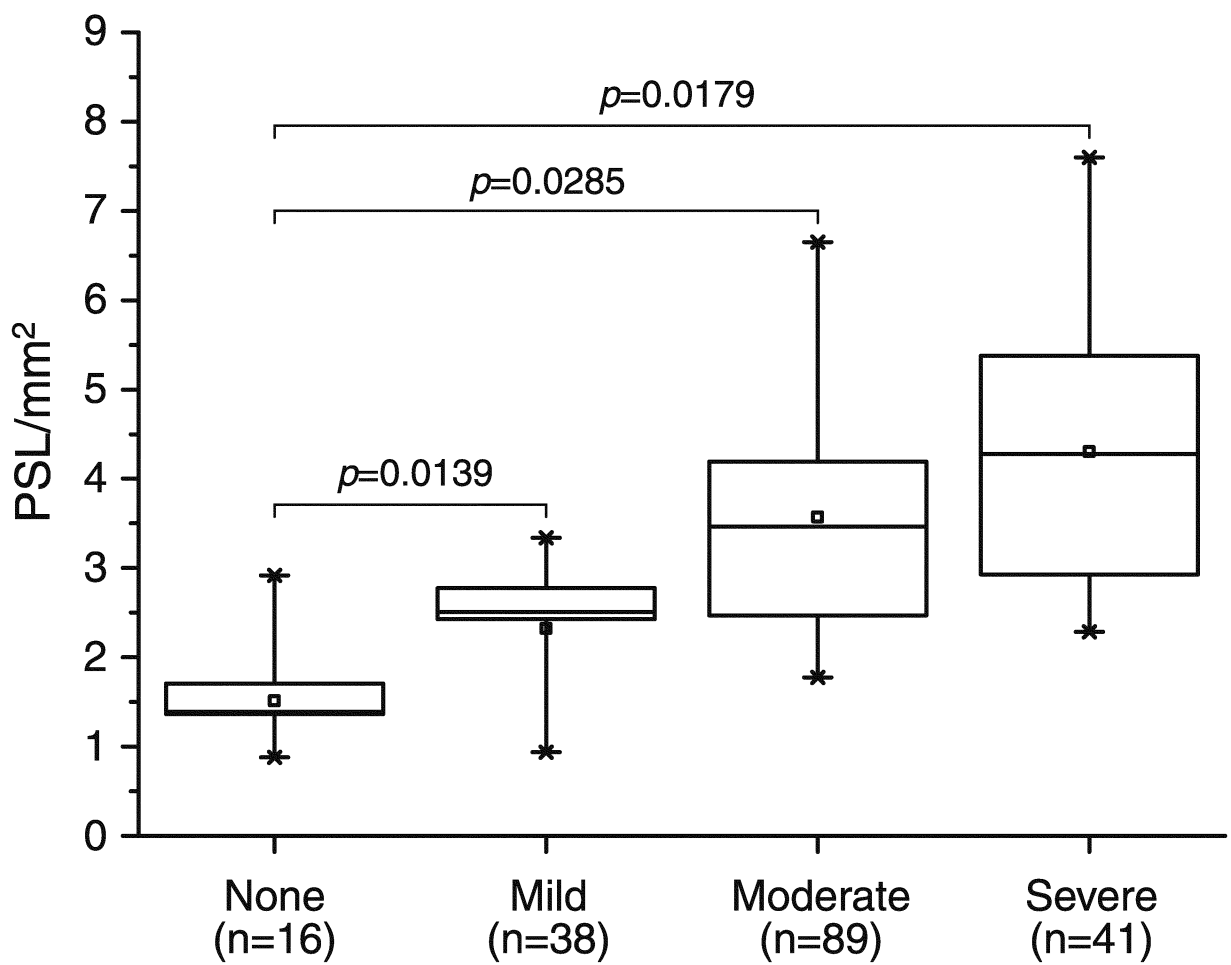
**Uptake of ^68^Ga-radioactivity and the degree of inflammation in atherosclerotic plaques**. Uptake of ^68^Ga-radioactivity in atherosclerotic plaques of LDLR*^-/-^*ApoB^100/100 ^mice in comparison to the degree of inflammation assessed on the basis of Mac-3 immunohistochemistry. None, no macrophages; Mild, occasional macrophages; Moderate, occasional and some groups of macrophages; Severe, abundant infiltration of macrophages. The ^68^Ga-radioactivity uptake results are expressed as PSL/mm^2^(mean ± SD), normalized against internal control tissue (muscle).

**Figure 3:**
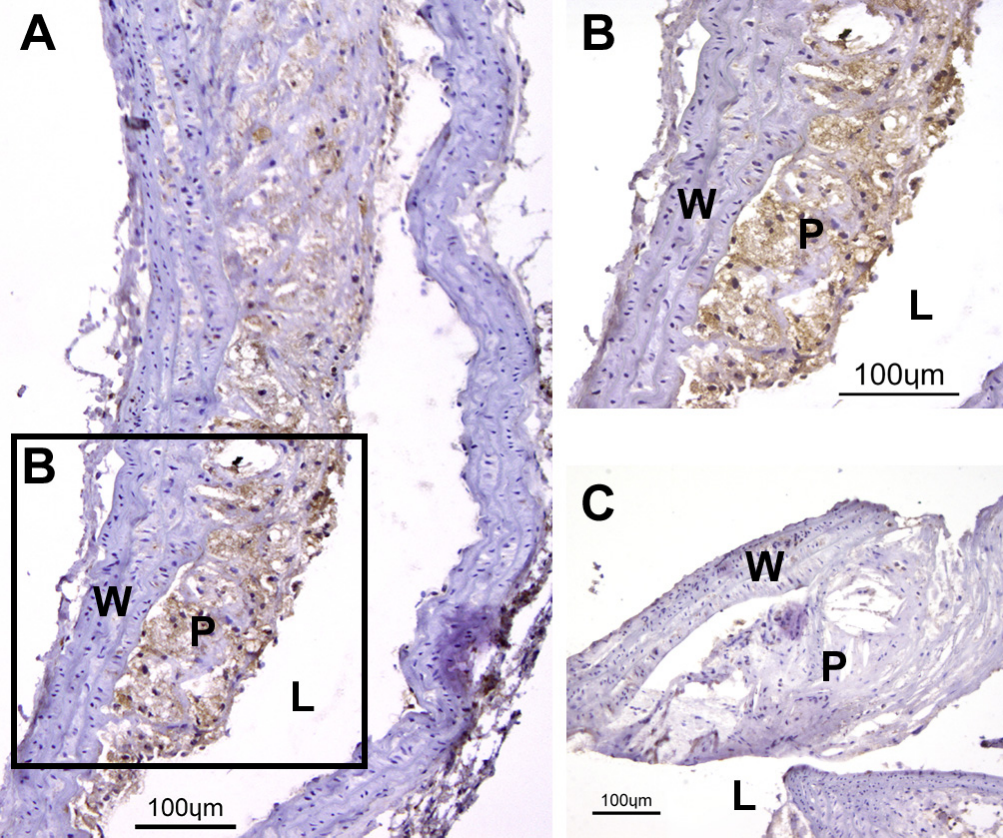
**Mac-3 immunostained atherosclerotic plaques**. (**A**) Representative Mac-3 immunostainings of severely inflamed plaque. Macrophages are shown in brown color. Adventitia and healthy vessel wall areas were Mac-3 negative. (B) An enlarged area rich in macrophages marked in (A). (C) Non-inflamed plaque with only scanty immunopositivity for Mac-3. L, lumen; P, plaque; W wall.

A total of 184 plaques obtained from four aortic cryosections of each LDLR*^-/-^*ApoB^100/100 ^mouse were analyzed and normalized against internal control tissue (muscle). According to the autoradiographic analysis, the uptake of ^68^Ga in plaques with different degrees of inflammation was 1.5 ± 0.7 PSL/mm^2 ^in non-inflamed plaques (*n *= 16), 2.3 ± 0.8 PSL/mm^2 ^in mildly inflamed plaques (*n *= 38), 3.6 ± 1.5 PSL/mm^2 ^in moderately inflamed plaques (*n *= 89), and 4.3 ± 1.9 PSL/mm^2 ^in severely inflamed plaques (*n *= 41). Figure 2 shows the co-localisation of the ^68^Ga-radioactivity uptake and degree of macrophage-rich areas in atherosclerotic plaques. Representative Mac-3 stainings of non-inflamed and severely inflamed plaques are shown in Figure 3.

### *In vivo *imaging

**Figure 4:**
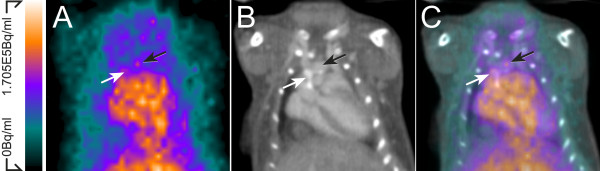
***In vivo *imaging**. (A) *In vivo *PET, (B) CT angiography, and (C) fused PET/CT images demonstrate the high ^68^Ga signal in the blood pool as seen in the aorta and heart. Calcified (white arrows) and non-calcified (black arrows), aortic arch and aortic root can be seen in contrast-enhanced CT angiography.

*In vivo *PET/CT imaging demonstrated high radioactivity in the blood pool (8.1 ± 2.2% IA/g, *n *= 3) at 3 h after ^68^Ga injection as seen in the aorta and heart of LDLR*^-/-^*ApoB^100/100 ^mouse (Figure 4). The *in vivo *uptake of ^68^Ga-radioactivity in the aortic arch and brachiocephalic artery was 6.4 ± 3.0 and 5.8 ± 2.5% IA/g, respectively.

## Discussion

Macrophages play a crucial role in the progression and development of atherosclerosis from early fatty-streak lesions to advanced atherosclerotic plaques [11]. Ruptured plaques, in particular, commonly contain high quantities of macrophages [3]. Therefore, the contents of macrophages may be an excellent imaging biomarker for the vulnerability of atherosclerotic plaques. The present results revealed that the ^68^Ga-radioactivity uptake in atherosclerotic plaques, especially in their macrophage-rich areas, was higher when compared to healthy vessel wall. This observation strengthens our conception that ^68^Ga-radioactivity accumulates at the site of inflammation.

^68^Ga radionuclide is readily available by elution from a ^68^Ge/^68^Ga generator with 0.1 M HCl in the form of ^68^Ga-chloride. However, after neutralization, ^68^Ga-chloride is hydrolyzed *in situ *or after administration *in vivo*. In general, ionic ^68^Ga^3+ ^is in the form of soluble anion called gallate, ^68^Ga(OH)^-^_4_, and/or insoluble colloidal particles ^68^Ga(OH)_3_. In the present study, the final ^68^Ga product contained 13% of colloidal forms of ^68^Ga. Uncharged gallium hydroxide or gallium nanoparticles are capable of penetrating the cell. Under the lower pH conditions, inside the cell, the material ionizes and becomes bound. Accordingly, accumulation into the liver, spleen, and bone marrow would be expected [12,13].

In blood, ^68^Ga-radioactivity can circulate also as a free ^68^Ga^3+ ^and mimic ferric ions. In spite of their physical similarities, gallium and iron behave differently *in vivo*. The major difference is the inability of gallium to be reduced *in vivo *wherefore gallium remains bound to iron-transport proteins and carrier molecules with the inability to interact with protoporphyrin IX to form heme. A high blood uptake of gallium is explained by its binding to iron-binding molecules; transferrin, lactoferrin, and ferritin. One way to facilitate the solubilization of ^68^Ga may be its chelation with citrate. For SPECT imaging, ^67^Ga is indeed administrated as citrate to avoid protein binding. Since citrate is only a weak chelator *in vivo*, ^67^Ga is rapidly released, hydrolyzed, and bound to transferrin and other iron-binding proteins [12,14-16], suggesting that chelation does not improve the uptake of the tracer. We have compared uptakes of ^68^Ga and ^68^Ga-citrate in rat osteomyelitis model (unpublished data), and ascertained that there were no differences between ^68^Ga and ^68^Ga-citrate uptakes in inflammation lesions.

In comparison to ^67^Ga-based SPECT, ^68^Ga-based PET may have some advantages, such as a better resolution for the detection of inflammation and a smaller radiation exposure for the subject. However, the blood clearance of ^68^Ga-radioactivity was slow as demonstrated by *ex vivo *measurement and by *in vivo *PET/CT imaging. Unfortunately, the relatively short half-life of ^68^Ga does not allow delayed imaging times for better blood clearance. In an inflamed lesion, ^67^Ga is accumulated in the areas rich in infiltrated macrophages. Interestingly, ^67^Ga is not accumulated at the sites of crowded neutrophils, suggesting that the polymorphonuclear leukocytes do not play a major role in the accumulation of ^67^Ga in inflammatory lesions [17].

In the present study, the plaque/wall ratio of ^68^Ga was 1.8 ± 0.2. As compared to 2-deoxy-2-[^18^F]fluoro-D-glucose (^18^F-FDG), we have measured the plaque/wall ratio of 2.7 ± 1.1 approximately in the same aged LDLR*^-/-^*ApoB^100/100 ^mice as used in the present study [18]. It has been shown that the clinical image qualities of ^68^Ga-tracers (*β *+ decay 97%, *Eβ *+ max 0.64 MeV) and ^18^F-tracers (*β *+ decay 97%, *Eβ *+ max 0.64 MeV) are not necessarily very much different from each other [19]. However, ^68^Ga has a higher kinetic energy compared to ^18^F, which could decrease the imaging spatial resolution, particularly when a small plaque lesion is imaged with a high resolution small PET/CT. Based on these earlier observations, ^18^F-FDG seems to be more promising PET tracer for imaging of atherosclerotic plaques than ^68^Ga.

Clinical observations indicate that in normal circumstances, the major proportion of the gallium retained in the body is localized in the bone and liver, and that inflammatory lesions alter this biodistribution, frequently causing decreased hepatic uptake [15]. In the present study, a difference in the uptake of ^68^Ga between the atherosclerotic mice and controls was found in bone and liver. The uptake of ^68^Ga-radioactivity in the whole aorta of atherosclerotic mice was higher than that in the control mice, but the difference was not significant. The uptake in the inflammatory plaques could partly explain the lower uptake of ^68^Ga in the liver of atherosclerotic mice. More likely this may be due to liver failure caused by high fat content, which can occur in older LDLR*^-/-^*ApoB^100/100 ^mice fed with a Western-type diet. Concerning the uptake of ^68^Ga in the bone, we assume that in the young control mice (aged 2.5 months), active bone remodeling may have contributed to the higher bone uptake as compared to the older LDLR*^-/-^*ApoB^100/100 ^mice (aged 10 to 11 months).

The accumulation of ^68^Ga-radioactivity in inflammatory lesions is complex, and the majority of ^68^Ga is probably delivered into the inflammatory lesions through the increased permeability at the site of atherosclerotic arteries. In the present study, accumulated ^68^Ga-radioactivity was detected in the macrophages-rich plaques suggesting that ^68^Ga accumulates at the site of inflammation. In addition to that, calcified areas of atherosclerotic plaques were also detected by ^68^Ga, which may be explained by the competitive binding of gallium ions to the Ca^2+ ^and Mg^2+ ^binding sites [17]. Since the calcified areas can be localized with CT, this observation is not limiting the use of ^68^Ga to distinguish macrophages-rich plaques and calcified plaques.

To some extent, gallium may also be taken up by atherosclerotic plaques due to its binding to the circulating transferrin and avidly to the transferrin receptors at the site of atherosclerotic arteries. Transferrin receptor expression has been shown to correlate positively with macrophage infiltration in human carotid atherosclerotic lesions [20]. Moreover, symptomatic patients with carotid atherosclerosis have significantly higher expression of transferrin receptors than asymptomatic patients [21]. However, although the uptake of ^68^Ga in inflamed atherosclerotic plaques was promising as measured by autoradiography, the slow blood clearance limits the use of ionic ^68^Ga for *in vivo *imaging of atherosclerotic plaques.

## Conclusions

Autoradiography analysis showed a moderate, but significantly higher ^68^Ga-radioactivity uptake in the aortic plaques of atherosclerotic mice, especially at the sites rich in macrophages, as compared to areas of healthy vessel wall. While the uptake of ^68^Ga-radioactivity was prominent in the atherosclerotic plaques of LDLR*^-/-^*ApoB^100/100 ^mice, the slow blood clearance limits the feasibility of ionic ^68^Ga for clinical imaging of atherosclerotic plaques especially in coronary arteries.

## Competing interests

The authors declare that they have no competing interests.

## Authors' contributions

JMUS contributed the conception and design, performed the experiments, analyzed and interpreted data and drafted the manuscript. IL performed the experiments, acquired, analyzed and interpreted data and participated in writing of the manuscript. HJS prepared the ^68^gallium and participated in writing of the manuscript. VJOL involved in designing the analysis and interpretation of data, and in drafting and revising the manuscript. PL involved in designing the experiments and revising the manuscript. SY-H, JK, and AR contributed the conception and design, critically contributed and revised the manuscript and enhanced its intellectual content. All authors have approved the final content of the manuscript.
